# FD-IDS: Federated Learning with Knowledge Distillation for Intrusion Detection in Non-IID IoT Environments

**DOI:** 10.3390/s25144309

**Published:** 2025-07-10

**Authors:** Haonan Peng, Chunming Wu, Yanfeng Xiao

**Affiliations:** 1 College of Computer Science and Technology, Zhejiang University, Hangzhou 310027, China; hnpeng@zju.edu.cn; 2 China Mobile Group Huzhou Co., Ltd., Huzhou 313098, China; xiaoyf@foxmail.com

**Keywords:** intrusion detection, IoT security, federated learning, knowledge distillation, Non-IID, feature selection

## Abstract

With the rapid advancement of Internet of Things (IoT) technology, intrusion detection systems (IDSs) have become pivotal in ensuring network security. However, the data produced by IoT devices is typically sensitive and tends to display non-independent and identically distributed (Non-IID) properties. These factors impose significant limitations on the application of traditional centralized learning. In response to these issues, this study introduces a novel IDS framework grounded in federated learning and knowledge distillation (KD), termed FD-IDS. The proposed FD-IDS aims to tackle issues related to safeguarding data privacy and distributed heterogeneity. FD-IDS employs mutual information for feature selection to enhance training efficiency. For Non-IID data scenarios, the system combines a proximal term with KD. The proximal term restricts the deviation between local and global models, while KD utilizes the global model to steer the training process of local models. Together, these mechanisms effectively alleviate the problem of model drift. Experiments conducted on both the Edge-IIoT and N-BaIoT datasets demonstrate that FD-IDS achieves promising detection performance across multiple evaluation metrics.

## 1. Introduction

Owing to its swift advancement, the extensive deployment of Internet of Things (IoT) across fields like smart cities, wearable devices, and healthcare has driven an exponential increase in the number of IoT devices [1,2]. According to Statista, the global number of connected IoT devices is projected to reach approximately 32.1 billion by 2030 [3]. This explosive growth has not only spurred technological advancements and economic development, but it has also significantly expanded the attack surface for cybersecurity threats. In particular, due to the resource-constrained, highly distributed, and diverse nature of IoT devices, attackers can exploit these vulnerabilities to launch various cyber threats [4]. Against this backdrop, ensuring the cybersecurity of IoT has become a critical issue for the stability of the global information society and economic development.

Intrusion detection systems (IDSs), as a crucial network security defense mechanism, have garnered extensive attention over the years. Especially driven by the swift progress of deep learning (DL), many researchers have employed DL-based models in IDSs to improve their effectiveness in areas like traffic analysis and anomaly identification [5,6,7]. Nevertheless, the applicability of traditional centralized learning (CL) in IoT scenarios is limited. CL generally depends on aggregating the vast data produced by IoT devices onto a centralized server to facilitate model training. This data-centralized processing poses significant threats to privacy-sensitive scenarios [8]. For instance, in intelligent healthcare systems, hospitals generate and store large volumes of highly sensitive patient data, including diagnostic images and treatment histories (particularly for critical conditions such as cancer [9]). Uploading such data to a centralized server for model training raises substantial privacy concerns—especially in the event of cyber-attacks that could lead to large-scale patient data breaches. Moreover, the transmission of high-resolution medical images and textual records imposes considerable pressure on both network bandwidth and system resources. Similar challenges have been observed in smart grid environments [10]. Smart meters deployed at the user end continuously record fine-grained electricity usage patterns, such as time-of-use distributions and appliance-level consumption frequencies. Studies have shown that such data can be exploited to infer household occupancy, activity patterns, and even family structure. If these data are centrally stored on utility provider servers, a successful cyber-attack or leakage may not only result in severe privacy violations, but also jeopardize the operational security and dispatch integrity of the power grid. To overcome these limitations, Google proposed the concept of federated learning (FL) in 2016 [11]. In contrast to conventional CL approaches, FL enables training processes to be conducted locally across distributed devices, transmitting only model parameters through communication networks instead of raw data. This approach effectively mitigates the risk of sensitive information leakage. Additionally, it reduces the communication overhead and energy consumption associated with large-scale data transmission [12].

Despite the substantial potential of FL in safeguarding data privacy and reducing communication costs, it faces significant challenges when applied to the non-independent and identically distributed (Non-IID) data in IoT environments [13,14,15]. Due to differences in hardware configurations, usage scenarios, and network conditions, the local data distributions of IoT devices are often highly imbalanced. Such data heterogeneity not only undermines the effectiveness of the global model, but it also introduces considerable drift between the global and local models [16]. Model drift mainly describes the gradual widening of differences between the global model and local models throughout the training process, leading to substantial parameter inconsistencies. This divergence arises from differences in the clients’ objective functions, leading to conflicting update directions that undermine the effectiveness and generalization ability of the aggregated model [17,18]. When the local gradient directions across clients are dispersed, the global updates struggle to balance the diverse objectives [19]. These challenges highlight the persistent bottlenecks in federated learning when addressing Non-IID data and when ensuring consistency between global and local models.

To overcome the challenges outlined earlier, this study introduces an FL-based IDS, named FD-IDS, which was designed for Non-IID IoT environments. Specifically, FD-IDS employs a deep neural network (DNN) as the classification model, leveraging its powerful nonlinear mapping capabilities to better capture complex intrusion patterns in IoT environments. To enhance feature quality, a mutual information (MI)-based feature selection method was introduced to filter high-dimensional traffic data, thereby reducing data dimensionality and computational overhead. In terms of the federated training strategy, FD-IDS integrates a knowledge distillation (KD) directly into the FL process. Unlike conventional approaches that typically design the distillation process as an offline or decoupled stage outside the training loop, FD-IDS embeds the distillation mechanism within each round of federated training. In this setup, the global model acts as a teacher, providing soft label supervision to the local student models during each communication round, thereby forming an interactive round-wise distillation process. This mechanism not only enhances the generalization ability of client models under Non-IID conditions, but it also improves the efficiency of information sharing among models. Furthermore, FD-IDS incorporates both the proximal term from the FedProx and the distillation loss function, establishing a dual regularization framework. Comprehensive experiments on both the Edge-IIoT and N-BaIoT datasets validated the effectiveness of FD-IDS. The primary contributions of this study are outlined as follows:

- This work presents an IDS utilizing FL, referred to as FD-IDS, which achieves intrusion detection in IoT environments while preserving data privacy.
- MI is employed for feature selection, enhancing feature quality, reducing redundancy in high-dimensional data, and improving model training efficacy and detection performance.
- By integrating a proximal term with KD, this paper mitigates the drift issue caused by Non-IID data through global and local collaborative optimization.
- The experiments were performed on both the Edge-IIoT and N-BaIoT datasets, with the results showing that FD-IDS delivers exceptional performance across various evaluation metrics.

The structure of this paper is as follows. Section 2 provides a review of the related work on IDSs. Section 3 details the proposed methodology. Section 4 presents the experimental results and analysis. Section 5 concludes this paper and discusses future research.

## 2. Related Work

In recent years, IDSs have emerged as a critical component of cybersecurity, drawing significant attention from both academia and industry. While traditional rule-based and signature-based IDS approaches have achieved success, their limitations become evident in the context of IoT [20]. Challenges, such as constrained device resources, diverse communication protocols, and dynamic network topologies, hinder their effectiveness. To address these issues, researchers have integrated machine learning (ML) and DL techniques, which leverage high-dimensional features extracted from massive network traffic, thereby improving detection rates and adaptability to unknown attacks [7,21]. Furthermore, to overcome challenges related to data privacy and the centralized processing of distributed data, technologies like FL have been applied in IoT-based IDSs, enhancing their feasibility and efficiency in practical scenarios [22,23]. Table 1 summarizes the recent research in the field of IDSs, encompassing representative works on CL and FL approaches.

Saurabh et al. [24] developed an long short-term memory network (LSTM)-based IDS (LBDMIDS) to detect sophisticated attacks in IoT networks. Their study incorporated both stacked LSTM and BiLSTM variants, and they were validated using the UNSW-NB15 and BoT-IoT datasets. Data preprocessing involved dimensionality reduction and standardization techniques. Experimental results showed that the stacked LSTM achieved 96.60% accuracy on the UNSW-NB15 dataset, while BiLSTM attained 96.41%. On the BoT-IoT dataset, both models excelled, achieving a remarkable 99.99% accuracy. Fatani et al. [25] introduced a feature extraction and selection method for IoT-based IDS, combining DL with the Aquila optimizer. The approach utilized convolutional neural networks (CNNs) for feature extraction and the Aquila optimizer for feature selection, reducing data dimensionality and enhancing classification performance. Evaluated on the KDD99, NSL-KDD, BoT-IoT, and CIC-IDS2017 datasets, the method was benchmarked against multiple metaheuristic algorithms, showcasing superior results.

Despite the advancements achieved through CL approaches in IDSs for IoT, real-world implementations face challenges, such as data privacy [8]. As a result, FL, a distributed ML framework, has been extensively employed in the design and optimization of IDSs. Ferrag et al. [26] developed the Edge-IIoTset cybersecurity dataset to support the performance evaluation of CL and FL models. The dataset is designed to support both IID and Non-IID data scenarios, enabling assessments in binary, six-class, and fifteen-class classification tasks. The experimental findings reveal that, in IID scenarios, the performance of the global model in FL is nearly identical to that of CL models. In Non-IID scenarios, FL enhances the overall performance of clients by efficiently aggregating their local models. Rashid et al. [27] introduced an IIoT IDS based on FL, leveraging the Edge-IIoTset dataset. Their study evaluated two DL classifiers—CNNs and recurrent neural networks (RNNs)—in CL and FL scenarios. The comparative analysis revealed that the RNN model achieved superior global detection accuracy under FL settings, particularly with Non-IID data. Using three clients, the RNN model achieved a global detection accuracy of 91.87%. Aouedi et al. [28] proposed F-BIDS, an FL-based IDS that utilizes decision trees (DTs) and random forests (RFs) as foundational classifiers. Metadata are created locally by users and sent to a central server through FL for training a global neural network (NN). Experiments on the Edge-IIoTset and InSDN datasets demonstrated the model’s efficacy. For a fifteen-class task on Edge-IIoTset, the global model achieved 89.91% accuracy after 10 training rounds. On the InSDN dataset, F-BIDS achieved a global model accuracy of 99.91% after 50 rounds, with the lowest client accuracy exceeding 99.70%. Nobakht et al. [29] proposed SIM-FED, which employs a lightweight 1D CNN. Unlike traditional 2D-CNN-based methods, SIM-FED reduces preprocessing time and computational overhead. Through hyperparameter optimization, the model achieves superior detection performance while minimizing resource demands. Utilizing the FedAvg strategy for global parameter aggregation, SIM-FED effectively integrates training results from multiple local models in distributed environments.

In FL, the issue of Non-IID data is widely regarded as a core challenge that significantly impacts model performance [14,15,30]. Effectively handling Non-IID data and mitigating the negative impact of data heterogeneity on model training has become a pivotal research direction for improving the performance of FL systems. Belarbi et al. [31] simulated the negative impact of Non-IID data on model performance by partitioning the ToN-IoT dataset based on target IP addresses, thereby replicating real-world network traffic scenarios. They proposed two FL architectures based on DNNs and deep belief networks (DBNs), and they conducted a comparative analysis of three aggregation methods. Additionally, they explored an initialization strategy based on pre-trained global models to further enhance performance. Experimental results demonstrated that FedProx and FedYogi exhibit greater stability under Non-IID data conditions, while pre-trained models effectively mitigate performance degradation caused by data heterogeneity, achieving an F1 score improvement of over 20% compared to randomly initialized models. Nugraha et al. [32] proposed an IDS that integrates FL and variational autoencoders (VAEs) for DDoS attack detection. The approach leverages VAEs on clients to learn local normal traffic features, while FedAvgM aggregates model parameters for efficient detection of malicious traffic. To address Non-IID data and data imbalance challenges, the framework incorporates dynamic client sampling, continuous model retraining, and the BCEWithLogitsLoss function to improve detection performance. Benameur et al. [33] proposed an IDS based on FL and KD. Utilizing the Edge-IIoTset dataset, they designed a distributed learning architecture centered on a teacher network and employed a lightweight student network for efficient inference. Their study compared the performance of CL and FL using three model architectures: DNNs, CNNs, and CNN-LSTM. In the FL scenario, the highest accuracy for the 15-class classification task was achieved by the CNN-LSTM model (82.4%), followed by the CNNs model (82.35%) and then the DNNs model (82.09%). Furthermore, the experimental results demonstrated that KD effectively enhanced model performance. This approach adopted a traditional offline distillation method, in which a pre-trained, complex teacher model is compressed into a lightweight student model, thereby addressing the challenges posed by limited computational resources.

The aforementioned related works have made significant contributions to the research on IDSs and have inspired this study. However, existing approaches that integrate FL with KD primarily focus on model compression and communication efficiency optimization. These methods typically adopt offline distillation or exhibit weak coupling with the federated optimization process, making it difficult to achieve synchronized convergence between global and local models during training. As a result, the issue of model drift under Non-IID conditions remains inadequately addressed. This paper proposes FD-IDS, an IDS tailored for Non-IID IoT environments. The proposed system was developed to tackle key challenges, including data privacy protection and the issue of distributed heterogeneity. FD-IDS employs a MI-based feature selection method to effectively reduce model complexity and computational overhead. In terms of optimization, FD-IDS introduces a dual-constraint collaborative design mechanism that integrates the proximal term with KD. Unlike conventional approaches that treat distillation as a post-training procedure or loosely connected component, FD-IDS embeds this mechanism directly into each round of federated training. This enables dynamic collaborative training guided by both parameter synchronization constraints and semantic consistency supervision. Experiments were conducted using the Edge-IIoT and N-BaIoT datasets to evaluate the performance of FD-IDS.

**Table 1:** Summary of the recent research on IDSs.

Ref.	Year	Dataset	Algorithm	Learning Strategy	Client	Aggregation Strategy	Data Distribution
[24]	2022	UNSW-NB15, BoT-IoT	LSTM	CL	**–**	**–**	**–**
[25]	2021	KDD99, NSL-KDD, BoT-IoT, CICIDS-2017	CNN	CL	**–**	**–**	**–**
[26]	2022	Edge-IIoTset	DT, RF, KNN, SVM, DNN	CL, FL	K = 5, 10, 15	FedAvg	IID, Non-IID
[27]	2022	Edge-IIoTset	CNN, RNN	CL, FL	K = 3, 9, 15	FedAvg	IID, Non-IID
[28]	2023	Edge-IIoTset, InSDN	DT, RF	CL, FL	K = 5, 10, 15	FedAvg	**Ø**
[29]	2024	IoT-23	CNN	CL, FL	K = 10, 15, 20	FedAvg, FedAvgM, FedMedian, FedProx	**Ø**
[31]	2023	TON-IoT	DNN, DBN	CL, FL	K = 10	FedAvg, FedProx, FedYogi	Non-IID
[32]	2024	CIC-DDoS2019	VAE	CL, FL	K = 6	FedAvgM	Non-IID
[33]	2024	Edge-IIoTset	CNN, DNN, CNN-LSTM	CL, FL	K = 4	FedAvg	**Ø**
Our method	**-**	Edge-IIoTset, N-BaIoT	FD-IDS	CL, FL	K = 9	FedProx	Non-IID

**–** indicates data are not applicable, whereas **Ø** indicates that the corresponding study did not mention this information.

## 3. Methodology

### 3.1. Overview

**Figure 1:**
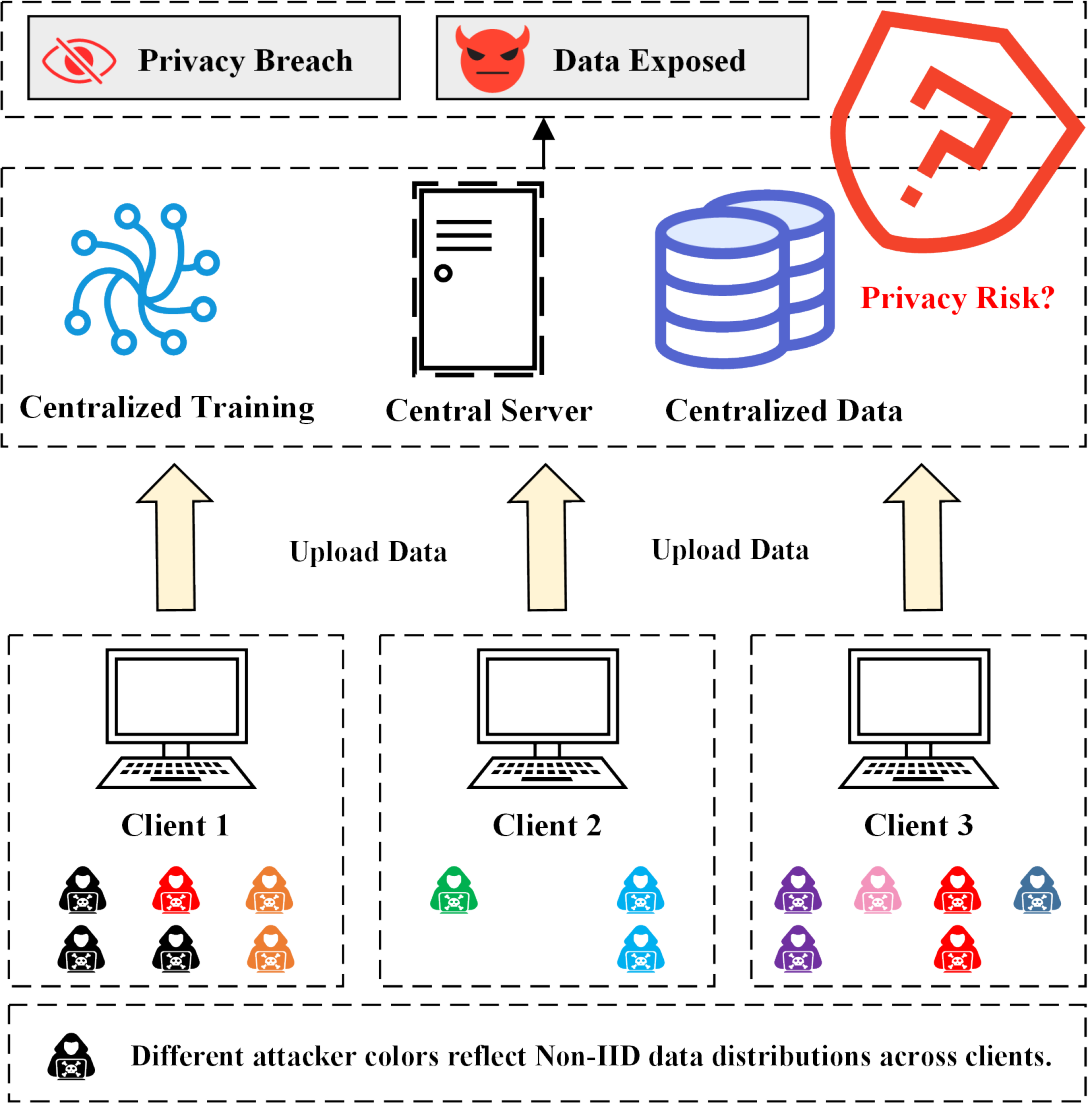
Data heterogeneity and privacy risk in a centralized intrusion detection architecture.

In IoT environments, the inherent Non-IID characteristics of data present considerable obstacles to the effectiveness of IDSs. Figure 1 illustrates a CL architecture in the context of IoT, where each client device uploads locally collected raw network traffic data to a central server for unified model training. The types and distributions of attacks vary across clients. Different colors and quantities of attacker icons represent class imbalance and sample diversity in local datasets, highlighting the inherent Non-IID characteristics of the data. Moreover, under this architecture, all sensitive data are collected and transmitted to the central server. In the event of a server compromise or data breach, users’ privacy would be exposed to significant security risks. To address these challenges, this paper proposes a novel intrusion detection method based on the FL, termed FD-IDS. The proposed method integrates a proximal term and KD mechanism to effectively mitigate the performance drift caused by Non-IID data while preserving data privacy. This approach enhances the overall detection performance of the system in heterogeneous environments.

To assess the effectiveness of FD-IDS in IoT environments, we utilized the publicly available Edge-IIoT and N-BaIoT datasets and performed data preprocessing. For the model architecture, a DNN was selected as the classifier for the IDS because of its strong feature extraction and learning capabilities, enabling precise identification of complex attack patterns.

**Figure 2:**
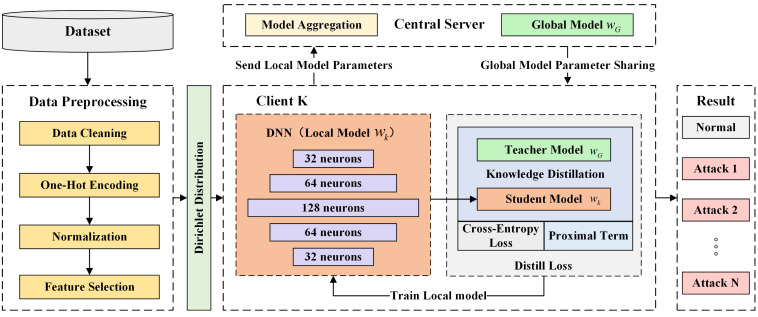
Methodology flowchart.

In the FL training phase, the FedProx aggregation algorithm was adopted. By incorporating a proximal term into the objective function, FedProx effectively constrains the extent of local model updates, thereby alleviating the model drift caused by Non-IID [34]. To further mitigate the drift issues resulting from data distribution discrepancies, this paper introduces a KD technique. By guiding the training process of local models with the global model, KD effectively transfers the global model’s knowledge to local models. This mechanism helps local models maintain consistency with the global model, thus reducing the performance drift induced by Non-IID data. It improves the generalization capacity of local models, enabling them to iteratively adjust deviations from the global model during training, ensuring a synergistic effect between local and global models in FL. Figure 2 illustrates the workflow of FD-IDS.

### 3.2. Dataset Description

In numerous studies, the development of IoT IDSs often relies on datasets collected from traditional network environments as the basis for training. However, there are significant differences between traditional networks and IoT environments in terms of communication protocols, device resource constraints, and traffic characteristics. This environmental heterogeneity can affect the applicability and detection performance of IDSs trained on traditional datasets when deployed in real IoT scenarios [35]. To overcome this limitation and better meet the practical needs of IoT environments, we employed the Edge-IIoT and N-BaIoT datasets for the experiments.

- The Edge-IIoT dataset is specifically designed for IoT environments and encompasses a diverse array of IoT devices, such as pH sensor meters and heart rate sensors, among others. This diversity enables the dataset to comprehensively reflect the variety of IoT devices and their traffic characteristics [26]. Moreover, the dataset contains over ten types of common attacks, covering a broad spectrum of potential security threats in IoT environments.
- The N-BaIoT dataset, widely used in IoT security research, comprises traffic data from various smart home devices, such as doorbells and monitors, providing strong device diversity [36]. The dataset includes several common attack types, effectively reflecting the anomalous traffic behavior of compromised devices in an IoT environment.

### 3.3. Data Preprocessing

To enhance the effectiveness of the model training, we performed data preprocessing on the datasets. First, we removed potentially detrimental features, such as “udp.port”, “ip.src_host”, and “ip.dst_host” [26]. Although these features, which contain information on IP addresses and port numbers, may contribute to data distinguishability, they pose significant security risks in real-world applications. For instance, attackers can evade detection by spoofing IP addresses or randomizing port numbers. Retaining such features may lead to models overly relying on specific network attributes, resulting in poor generalization performance in practical scenarios [37]. Next, we removed samples containing missing or infinite values. We also applied one-hot encoding to convert nominal categorical features into numerical representations. Since IoT devices generate data with diverse feature types and ranges across different sensors, we standardized the feature values using Z-score normalization to ensure consistent scaling.

After the aforementioned data preprocessing, the feature dimensions of the Edge-IIoT and N-BaIoT datasets expanded to 95 and 115, respectively. Although high-dimensional features facilitate the representation of detailed information and complex patterns in data, they also pose challenges, such as the curse of dimensionality, which can degrade model training efficiency and significantly increase computational overhead. To address these challenges, we employed the MI method. MI quantifies the shared information between each feature and the target variable, enabling the evaluation of features that significantly contribute to model performance while identifying irrelevant or redundant features. We calculated the MI score for each feature in relation to the target variable, and we then ranked them according to their scores. For the Edge-IIoT and N-BaIoT datasets, the top 25 and 71 features, respectively, were selected as inputs for the model training. The formula for MI is as follows:

(1) $$I\left(V_{x};V_{y}\right)=E\left(V_{x}\right)+E\left(V_{y}\right)-JE\left(V_{x},V_{y}\right),$$

where $V_{x}$ and $V_{y}$ represent two random variables, $E(V_{x})$ denotes the entropy of $V_{x}$, $E(V_{y})$ represents the entropy of $V_{y}$, while $JE(V_{x},V_{y})$ denotes the joint entropy between $V_{x}$ and $V_{y}$.

### 3.4. Model Architecture

**Figure 3:**
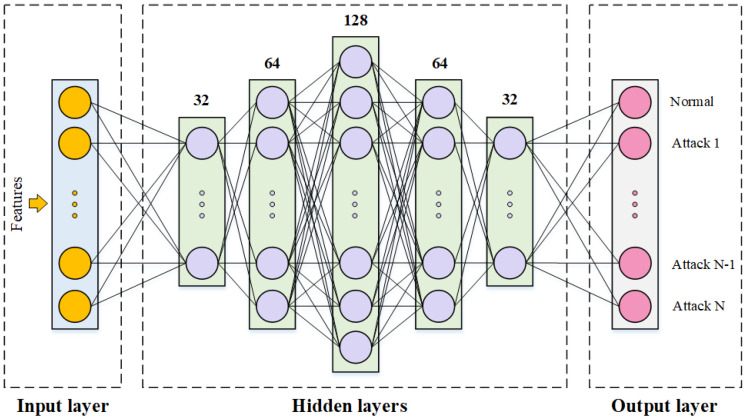
DNN model structure.

In the construction of IDSs within FL frameworks, the choice of the base classification model plays a pivotal role in determining the overall system performance. DNNs, with their superior feature extraction capabilities and ability to model complex nonlinear patterns, have emerged as an ideal choice for achieving efficient classification [38]. The DNN architecture employed in this study is depicted in Figure 3. It consists of an input layer, five hidden layers, and an output layer. The hidden layers are symmetrically arranged, with the number of neurons set to 32, 64, 128, 64, and 32, respectively. To enhance the model’s nonlinear representational capacity, each hidden layer incorporates the ReLU activation function. A softmax function is applied at the output layer to convert the final features into a probability distribution, allowing for accurate predictions in multi-class classification tasks. To optimize the model parameters, the Adam optimizer was chosen with a learning rate of 0.001.

### 3.5. Federated Learning Process with Knowledge Distillation

**Figure 4:**
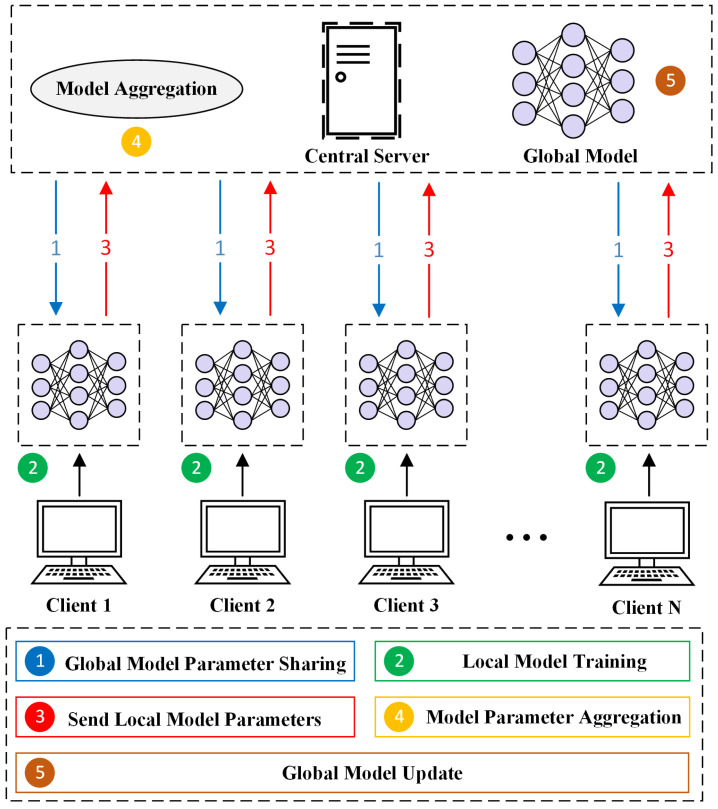
FL architecture.

FL is an emerging distributed machine learning framework aimed at enabling collaborative model training while ensuring data privacy and security [11]. Unlike traditional CL methods, FL eliminates the need for participants to send their data to a central server. Each participant retains data locally and sends locally generated model updates to a central server for aggregation, thereby constructing a global model. Based on the characteristics of data distribution, FL can be categorized into horizontal FL (HFL), vertical FL (VFL), and federated transfer learning (FTL) [34,39]. HFL is applicable in scenarios where participants share a common feature space but have distinct data instances, which makes it the most commonly used form of FL. VFL is suitable for scenarios where participants have the same data instances but operate within different feature spaces. FTL addresses situations where both sample and feature spaces partially overlap, focusing on facilitating knowledge transfer across domains. This study focused on HFL and explores its application in constructing efficient IDSs. Figure 4 illustrates the architecture of FL. This study primarily focused on the performance challenges posed by Non-IID data. Accordingly, the experimental design was based on the following key assumption: both the central server and the clients in the FL framework are considered trusted parties, that is, neither party intentionally alters the data nor injects harmful data to undermine the performance of the global model.

#### 3.5.1. Federated Learning Framework and Aggregation Strategies

In FD-IDS, the system architecture primarily consists of a central server and multiple client devices. The central server initializes the parameters $W_{G}^{0}$ of the global model, which are subsequently sent to the participating clients. Each client trains locally on its dataset $D_{k}={\left\{(x_{i},y_{i})\right\}}_{i=1}^{n_{k}}$, where $n_{k}$ denotes the sample size of client *k*, $x_{i}$ refers to the features of the *i*-th sample, and $y_{i}$ indicates the associated label. Data privacy is preserved using local computation, ensuring that clients do not transmit raw data to the central server or to other clients, thereby reducing the risk of data breaches [12]. Clients independently optimize their local objective functions to generate updated model parameters $w_{k}^{i+1}$. These updates are subsequently sent to the central server, where they are aggregated using a aggregation algorithm to generate the global model parameters $w_{G}^{i+1}$.

A widely adopted aggregation algorithm is FedAvg [11]. The core idea of FedAvg is to compute a weighted average of the model updates uploaded by the clients based on their respective sample sizes, ensuring that the global model reflects the diversity of distributed data effectively. The equation for FedAvg is given as follows:

(2) $$w_{G}^{t+1}=\sum_{k=1}^{K}\frac{n_{k}}{n}w_{k}^{t+1},$$

where *K* represents the total count of clients involved in the present training round, $n_{k}$ is the sample size of client *k*, and $n=\sum_{k=1}^{K}n_{k}$ refers to the aggregate number of samples from all clients. This weighting mechanism accounts for the contribution of each client to the global model.

Nevertheless, in practical settings, the data distribution across clients is often Non-IID, which poses significant challenges to FedAvg, such as biased model aggregation and accumulated drift during training, ultimately degrading the performance of the global model [16,40]. To address these challenges, this study adopted the FedProx algorithm as an optimization strategy [34]. FedProx incorporates a proximal term $L_{\mathrm{proximal}}$ into the local objective function, which limits the deviation of local models from the global model to reduce the optimization drift caused by Non-IID data. The proximal term is defined as follows:

(3) $$L_{\mathrm{proximal}}=\frac{\mu }{2}{∥w_{k}-w_{G}^{t}∥}^{2},$$

where ${∥w_{k}-w_{G}^{t}∥}^{2}$ represents the squared Euclidean distance between the parameters of the local model $w_{k}$ and the current global model $w_{G}^{t}$, which quantifies the degree of deviation of the local model from the global model. The parameter μ serves as a regularization parameter to control the impact of the proximal term on the optimization process. A larger μ imposes stricter constraints on local model updates, reducing the drift caused by Non-IID data, while a smaller μ allows greater flexibility in local model updates. In this study, μ was set to 0.01.

Compared to the FedAvg algorithm, FedProx achieves more balanced aggregation across heterogeneous client data. Particularly in practical IoT deployments, where attack behavior patterns captured by different sensors can vary significantly, FedProx enhances overall system consistency by introducing a proximal term that constrains local model divergence. This mechanism effectively mitigates the negative impact of extreme updates from specific types of devices on the performance of the global model.

#### 3.5.2. Federated Learning Optimization Based on Knowledge Distillation

The issue of Non-IID data remains one of the core challenges limiting model performance in FL. In IoT environments, heterogeneous devices are exposed to varying types of attacks. If local models are aggregated without appropriate constraints, the resulting global model may fail to detect certain attack types effectively. While the FedProx algorithm reduces the divergence between client models and the global model by adding a proximal term, its performance may still deteriorate or undergo drift in situations with extreme data distributions. To overcome these challenges, this paper introduces an innovative approach that incorporates KD into the FL framework. This method leverages continuous guidance from the global model to the client models, mitigating the model drift in Non-IID scenarios and, thereby, improving the detection accuracy of diverse IoT attacks while enhancing the overall security performance of the system.

**Figure 5:**
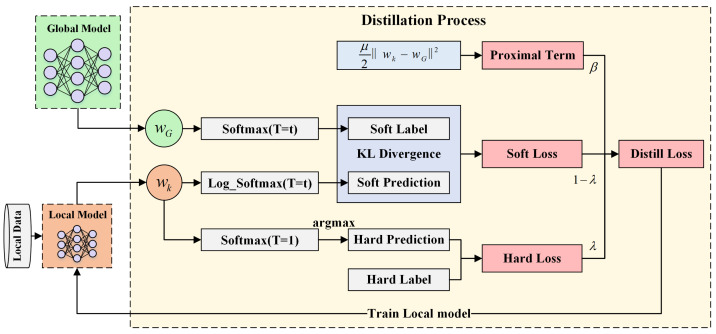
Distillation process.

KD, originally introduced as a model compression technique, relies on the concept of transferring information from a teacher model to a student model to enhance the student’s performance [41,42]. In the proposed FL framework, the global model functions as the teacher model, while the local models on the clients serve as the student models. Through KD, the global model continuously provides soft target distributions to the client models during each training round. These soft targets not only offer richer supervision compared to hard labels, but also effectively bridge the discrepancies caused by Non-IID between the global and local models. This mechanism ensures that the training of client models is consistently aligned with the global model’s guidance, preventing model drift due to local data imbalances. The distillation process is illustrated in Figure 5.

KD transfers probabilistic information between the teacher model (global model) and the student model (client model) by calculating the Kullback–Leibler (KL) divergence between their respective outputs [43]. The distillation loss is defined as follows:

(4) $$L_{\mathrm{soft}}=T^{2}\cdot \mathrm{KL}\left(\mathrm{softmax}\left(\frac{Z_{t}}{T}\right)\;∥\;\mathrm{softmax}\left(\frac{Z_{s}}{T}\right)\right),$$

where $Z_{t}$ and $Z_{s}$ represent the logits of the teacher model (global model) and the student model (client model), respectively. The parameter *T* is a temperature factor that smooths the output probability distribution. By increasing the temperature *T*, the student model can capture finer-grained probabilities from the global model, enhancing its generalization capabilities. In this study, *T* was set to 3.

Under the FedAvg aggregation strategy, the total distillation loss is defined as follows:

(5) $$L_{\mathrm{distill}}=\lambda \cdot L_{\mathrm{hard}}+\left(1-\lambda \right)\cdot L_{\mathrm{soft}},$$

where $L_{\mathrm{hard}}$ represents the hard-label loss, and $L_{\mathrm{soft}}$ is the soft-label loss. The weighting parameter λ balances the contributions of the hard-label and soft-label losses, and it was set to 0.5 in this study.

For the FedProx aggregation strategy, a proximal term was incorporated into the FedAvg framework to constrain the divergence between client models and the global model parameters. Combining this proximal term with KD, the client optimization objective is defined as follows:

(6) $$L_{\mathrm{distill}}=\lambda \cdot L_{\mathrm{hard}}+\left(1-\lambda \right)\cdot L_{\mathrm{soft}}+\beta \cdot L_{\mathrm{proximal}},$$

where $L_{\mathrm{proximal}}$ denotes the proximal term from FedProx and β is its weighting factor, which was set to 0.1 in this study.

By incorporating distillation loss, the global model can further guide client models through soft targets, ensuring that their training process remains aligned with the optimization direction of the global model and minimizing model drift to the greatest extent. The federated distillation process, which integrates the proximal term, is illustrated in Algorithm 1.
**Algorithm 1** FL with FedProx and KD.**Require:** Number of communication rounds *R*, total clients *K*, local dataset $D_{k}$, local  epochs *E*, learning rate η, loss weights λ and β
**Ensure:** Final global model $w_{G}^{R}$
  1: **Server:**
  2: Initialize $w_{G}^{0}$
  3: **for** 
t=1,2,…,R **do**
  4:     Set m=K (all clients participate)
  5:     **for** each client k=1,2,…,K (in parallel) **do**
  6:        Send $w_{G}^{t}$ to client *k*
  7:        Receive $w_{k}^{t+1}$ from client *k*
  8:     **end for**
  9:     Aggregate local models to update the global model:$w_{G}^{t+1}=\sum_{k=1}^{K}\frac{n_{k}}{n}w_{k}^{t+1}$
10:  **end for**
11:  Return $w_{G}^{R}$
12:  **Client:**
13:  **Function** ClientUpdate($w_{G}^{t},D_{k}$):
14:  Initialize $w_{k}=w_{G}^{t}$
15:  **for** local epoch e=1,2,…,E **do**
16:     **for** batch $b\subseteq D_{k}$ **do**
17:        Compute loss function:$L_{\mathrm{distill}}=\lambda \cdot L_{\mathrm{hard}}+\left(1-\lambda \right)\cdot L_{\mathrm{soft}}+\beta \cdot L_{\mathrm{proximal}}$
18:        Update $w_{k}$:$$w_{k}\leftarrow w_{k}-\eta \nabla L$$
19:     **end for**
20:  **end for**
21:  Return $w_{k}$ to the server


## 4. Experiments and Evaluation

### 4.1. Experimental Setup

To assess the model’s performance, each dataset was divided into training and testing sets with an 80/20 ratio, and the experiments were conducted within a FL framework. The FL environment consisted of nine clients. In this study, a total of nine clients was selected to simulate the FL environment based on the following considerations: First, a number of existing studies have also adopted a limited number of clients for federated learning experiments [31,32,33]. Second, this scale effectively emulates typical small-scale IoT deployment scenarios—such as industrial control systems and factory edge node networks. Third, FL experiments generally involve independent training of multiple local models and frequent communication rounds, resulting in substantial computational and memory overhead. Given the current hardware constraints, this client configuration helps ensure the stability of the experiments. To further evaluate the scalability of the proposed method in larger distributed environments, future work will expand the number of participating clients and conduct more comprehensive experimental studies.

To simulate the Non-IID nature of data, this study employed the Dirichlet distribution to partition the training dataset. This approach is widely adopted in FL research for modeling data heterogeneity across clients [44,45].

Let *C* be the total number of classes. Then, for the *k*-th client, the label distribution vector is defined as follows:

(7) $$p^{\left(k\right)}=\left(p_{1}^{k},\ldots ,p_{C}^{k}\right)∼\mathrm{Dir}\left(\theta \right),$$

where θ>0 denotes the concentration parameter, which controls the degree of similarity among client distributions. Specifically, the following applies:

- When θ→∞, the class distributions across clients become nearly identical, approximating an IID scenario;
- When θ=1, the distributions exhibit moderate variability around a uniform distribution, representing low Non-IID;
- When θ≪1 (e.g., 0.1), the distributions become highly sparse, with each client predominantly containing only a few classes, indicating high Non-IID.

In this study, we designed two data heterogeneity settings to reflect real-world IoT environments:

(1) **A low-Non-IID scenario,** where the Dirichlet concentration parameter is set to θ=1;
(2) **A high-Non-IID scenario**, where θ=0.1.

This design effectively simulates the class imbalance and label sparsity commonly encountered in FL, providing a representative environment for subsequent performance evaluation.

During the FL process, a synchronous communication mechanism was adopted to ensure that the global model was updated only after all clients had completed their local training. Each client conducted two epochs of local model training prior to synchronizing with the central server to upload their updates. The global model was optimized through 40 rounds of communication. We employed a grid search approach to determine the optimal combination of model hyperparameters. During this process, we referred to existing studies [18,27,29,34] and established a reasonable set of hyperparameter candidates based on commonly used empirical ranges. The complete set of candidate hyperparameters is presented in Table 2. After hyperparameter optimization, the final configuration adopted in this study was achieved, as detailed in Table 3.

### 4.2. Evaluation Metrics

To comprehensively and scientifically evaluate the performance of FD-IDS, selecting appropriate evaluation metrics is particularly crucial. Well-chosen metrics not only quantify the classification capability of the model effectively, but also reveal the system’s applicability in real-world scenarios from multiple perspectives. This study utilized a confusion matrix, as shown in Table 4, to describe classification outcomes. Based on this, the following four key evaluation metrics were defined.

(8) $$Accuracy=\frac{TP+TN}{TP+TN+FP+FN},$$

(9) $$Precision=\frac{TP}{TP+FP},$$

(10) $$Recall=\frac{TP}{TP+FN},$$

(11) $$F1-score=2\times \frac{Precision\times Recall}{Precision+Recall},$$

(12) $$\mathrm{FPR}=\frac{FP}{FP+TN},$$

(13) $$\mathrm{FNR}=\frac{FN}{FN+TP}.$$

### 4.3. Experimental Results and Analysis

#### 4.3.1. FD-IDS Performance Evaluation

FD-IDS was first assessed on the Edge-IIoT dataset across varying degrees of Non-IID conditions. Table 5 summarizes the outcomes of the experiments. In the low-Non-IID scenario, after the first communication round, the classification accuracy of the best-performing client reached 91.94%, while the worst-performing client achieved 88.75%. The global model accuracy was 85.27%. Despite the relatively low degree of data heterogeneity, performance discrepancies among clients were still observed. This indicates that, even in low-Non-IID conditions, the data distribution across clients is not entirely IID and a certain degree of imbalance persists. In contrast, the high-Non-IID scenario exhibited more pronounced performance disparities. After the first communication round, the best-performing client achieved an accuracy of 86.08%, whereas the worst-performing client had an accuracy of only 7.32%. The global model accuracy in this scenario was 77.12%.

Nonetheless, with a rise in communication rounds, the performance of both the clients and the global model improved progressively. During 40 communication rounds in the low-Non-IID scenario, the best-performing client’s accuracy increased to 94.65% and the worst-performing client’s accuracy reached 92.50%. The global model accuracy further improved to 94.82%. Similarly, in the high-Non-IID scenario, the best-performing client’s accuracy improved to 93.50%, the worst-performing client’s accuracy reached 88.25%, and the global model accuracy increased to 93.86%. In both scenarios, the performance gap between clients was significantly reduced and the consistency of the model across clients was enhanced. Furthermore, the global model’s performance improved.

To further evaluate the adaptability of FD-IDS under varying Non-IID data distributions, we conducted additional experiments using the N-BaIoT dataset. As shown in Table 5, the results on this dataset exhibit a degree of consistency with those obtained on the Edge-IIoT dataset. In the low-Non-IID scenario, after the first communication round, the classification accuracy of the best-performing client reached 72.87% while the worst-performing client achieved 55.10%, and the global model attained an accuracy of 53.21%. Under high-Non-IID conditions, performance further deteriorated, with the best client achieving 55.85% accuracy, the worst client achieving only 10.38%, and the global model reaching 38.58%. By Round 40, the accuracies of the best and worst clients in the low-Non-IID scenario rose to 87.69% and 86.38%, respectively, while the global model achieved 87.70%. In the high-Non-IID scenario, the accuracies increased to 83.25%, 78.45%, and 83.81%, respectively. These experimental results demonstrate that FD-IDS, through multiple communication rounds and iterative model updates, can effectively enhance the performance of client models while narrowing the performance gap among clients.

#### 4.3.2. Performance Comparison of Different Distillation Hyperparameter Settings and KD Intervals

**Figure 6:**
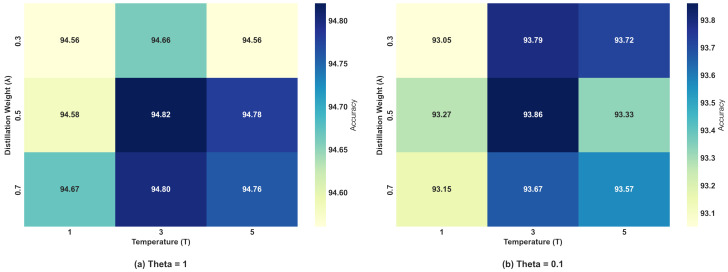
Accuracy under different distillation hyperparameter settings (Edge-IIoT).

**Figure 7:**
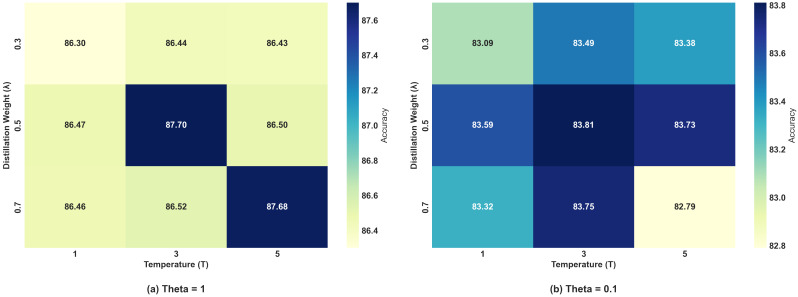
Accuracy under different distillation hyperparameter settings (N-BaIoT).

In FD-IDS, the setting of distillation-related hyperparameters, particularly the distillation weight (λ) and temperature (*T*), plays a critical role in determining the final model performance. To further optimize the distillation process, we conducted a systematic analysis of various candidate combinations of λ and *T*, as listed in Table 2. Experiments were performed on the Edge-IIoT and N-BaIoT datasets to evaluate how different hyperparameter settings influence model accuracy. The results, as illustrated in Figure 6 and Figure 7, highlight the accuracy variations under different settings.

As shown in Figure 6, on Edge-IIoT, the combination of λ = 0.5 and *T* = 3 yielded the highest accuracy (94.82%) in the low-Non-IID scenario, outperforming all other tested configurations. As *T* increased beyond this value, a slight degradation in accuracy was observed, suggesting that excessively high temperatures may lead to less stable knowledge transfer during the distillation process, thereby impacting the model’s overall performance. A similar trend was evident in the high-Non-IID scenario, where the same configuration (λ = 0.5, *T* = 3) again achieved the highest accuracy of 93.86%. As shown in Figure 7, on N-BaIoT, when θ=1, the combination of λ = 0.5 and *T* = 3 achieved the highest accuracy, reaching 87.70%. This configuration stood out among all tested hyperparameter settings, indicating its excellent performance in this scenario. When θ=0.1, the same combination also demonstrated the best performance, achieving an accuracy of 83.81%, which was slightly higher than that of the other configurations.

Overall, the comparison results across both datasets exhibited similar trends, with the combination of λ = 0.5 and *T* = 3 achieving the best accuracy in the different Non-IID scenarios. This further validated the effectiveness of this hyperparameter configuration across different datasets.

To further investigate the impact of KD intervals on the performance of FD-IDS, we designed a comparative experiment to validate the effectiveness of performing KD after each communication round in improving FD-IDS performance. In the experiment, three different KD intervals were defined:

- **Periodic KD**: KD is performed after every 8 communication rounds;
- **End-of-Training KD**: KD is performed in Rounds 31 to 40;
- **Round-wise KD**: KD is performed immediately after each communication round.

Table 6 presents the experimental results that were obtained using Edge-IIoT. In the low-Non-IID scenario, the accuracy of round-wise KD (94.82%) slightly outperformed that of periodic KD (94.71%) and end-of-training KD (94.70%). In the high-Non-IID scenario, the performance advantage of round-wise KD became even more apparent, with an accuracy of 93.86%, which was higher than periodic KD (93.21%) and end-of-training KD (92.97%). Table 7 presents the experimental results that were obtained using N-BaIoT. When θ=1, round-wise KD achieved an accuracy of 87.70%, which was higher than both periodic KD (86.28%) and end-of-training KD (86.25%). When θ=0.1, round-wise KD still outperformed the other two strategies, with an accuracy of 83.81%, surpassing periodic KD (81.53%) and end-of-training KD (80.72%).

Overall, the experimental results demonstrate that round-wise KD can effectively enhance the performance of FD-IDS under different Non-IID scenarios.

#### 4.3.3. Effectiveness of the Knowledge Distillation in FD-IDS

To assess how KD influences the performance of FD-IDS under Non-IID data scenarios, we designed comparative experiments incorporating and omitting KD based on the FedProx aggregation strategy. The results obtained with Edge-IIoT are presented in Table 8. In the case of a low-Non-IID scenario, the performance improvement from KD was slight. Specifically, the classification accuracy increased from 94.74% to 94.82%, and the F1 score improved from 94.17% to 94.35%. This slight enhancement can be attributed to the relatively minor data distribution differences among clients in low-Non-IID conditions, leading to more consistent model update directions and a more stable global aggregation process. In this context, even without KD, the FedProx effectively facilitated model convergence and performance improvement. Furthermore, the false positive rate (FPR) decreased from 0.14% to 0.13%, while the false negative rate (FNR) dropped from 5.26% to 5.18%. It is worth noting that, with respect to IDS, the impact of FNR is typically more critical than that of FPR. A FP indicates that benign traffic has been misclassified as an attack, which may lead to unnecessary resource consumption or degraded user experience, but the consequences are generally manageable. In contrast, a FN means that actual malicious traffic has gone undetected, allowing the attack to bypass the defense system, potentially resulting in severe consequences.

In contrast, under the high-Non-IID scenario, the benefits of KD were more pronounced. The classification accuracy improved from 91.89% to 93.86%, and the F1 score increased from 90.71% to 92.70%. This advantage stems from the unique adaptability of KD in heterogeneous data environments. Given the substantial data distribution divergence across clients, local models are prone to converging to local optima, and aggregation suffers from inconsistencies in client update directions, exacerbating global model drift. In such cases, KD, by introducing the global model as a teacher model, provides local models with soft label information rich in generalization capabilities. These soft labels convey the decision knowledge and prediction confidence information from the global model, reflecting the generalization capabilities formed through collaborative learning across multiple clients. Additionally, they provide supplementary training guidance for minority class samples, effectively mitigating the performance degradation issues caused by data heterogeneity. Moreover, the global model’s supervision signal, incorporated through KD during local training, steers the learning direction of local models closer to the global optimum, reducing the extent of model drift. It is noteworthy that, while KD enhances model performance, it also introduces additional computational overhead, primarily due to the necessity of calculating soft labels and the KL divergence loss during the distillation process, thereby increasing the computational burden on local devices. In terms of other metrics, the FNR decreased significantly from 8.11% to 6.14%. Although the FPR experienced a slight increase from 0.18% to 0.21%, it remained at a very low level overall.

**Figure 8:**
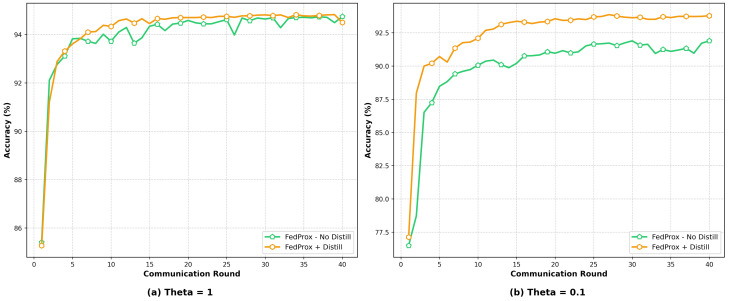
Accuracy comparison of an IDS with and without KD under different Non-IID scenarios on Edge-IIoT.

Figure 8 illustrates the accuracy trends of the IDS models with and without KD after each communication round under both low- and high-Non-IID scenarios. When θ=1, due to the minor differences in data distributions, the issue of model drift is relatively mild. As a result, the models with and without KD exhibited similar convergence trends. During the early stages of training, both approaches demonstrated comparable convergence speeds with steadily increasing accuracy. As the number of training rounds increases, both accuracies eventually stabilized, with the distilled model achieving only a slight improvement over the non-distilled model. When θ=0.1, the extreme heterogeneity in data distribution exacerbated the training bias of the local models and intensified the global model drift. As depicted in Figure 8, the model incorporating KD exhibited a faster accuracy increase during the early training stages and consistently outperformed the non-distilled model as training progressed. Ultimately, the distilled model achieved higher final accuracy, demonstrating the positive effect of KD in mitigating the challenges posed by Non-IID data.

In addition, we conducted experiments on the N-BaIoT dataset, with the results presented in Table 9. When θ=1, the performance improvement brought by KD was relatively limited. The accuracy increased slightly from 87.56% to 87.70%, the F1-score improved from 83.38% to 83.52%, the FNR decreased from 12.43% to 12.30%, and the FPR showed a marginal reduction from 1.91% to 1.90%. In contrast, when θ=0.1, KD yielded more notable performance gains. The accuracy improved from 81.51% to 83.81%, the F1-score rose from 77.17% to 78.24%, the FNR decreased from 18.49% to 16.19%, and the FPR remained unchanged. Notably, the use of KD introduced additional computational overhead.

#### 4.3.4. Impact of Different Aggregation Strategies on FD-IDS Performance

To assess how various aggregation strategies influence the performance of FD-IDS, we carried out experiments under both high- and low-Non-IID conditions. Table 10 presents the experimental results obtained on Edge-IIoT. When θ=1, the performances of FedAvg and FedProx were very close, with FedAvg achieving an accuracy and F1-score of 94.73% and 94.33%, respectively. This was, primarily, because the degree of data distribution heterogeneity among clients is relatively minor when θ=1. Although some Non-IID characteristics remained, the overall distribution differences were limited, thereby ensuring a higher degree of consistency in the model update directions. Under such conditions, FedAvg is able to effectively integrate client updates through weighted averaging, while the regularization constraints introduced by FedProx, although beneficial for mitigating Non-IID issues, offer only marginal improvements when data distribution differences are small. Consequently, the performance gain of FedProx in the low-Non-IID scenario was not significant. In this scenario, both aggregation methods yielded an identical FPR of 0.13%, while FedProx slightly reduced the FNR from 5.27% to 5.18%.

When θ=0.1, both FedAvg and FedProx exhibited performance degradation, indicating that highly Non-IID data pose greater challenges to FL. The pronounced distribution differences among clients exacerbated the inconsistency of the model update directions, thereby limiting the global model’s generalization capability across different clients. However, compared to FedAvg, FedProx demonstrated a smaller performance decline, achieving an accuracy and F1-score of 93.86% and 92.70%, respectively, thus outperforming FedAvg. This advantage was attributed to FedProx’s proximal term, which effectively constrains the excessive deviations of local models from the global model, thereby alleviating the model drift caused by Non-IID data. As a result, FedProx is able to maintain better global model performance even when client data distributions are highly heterogeneous. Moreover, FedProx reduced the FNR from 7.01% to 6.14% under high-Non-IID conditions. Although its FPR increased slightly from 0.18% to 0.21%, it remained at a low level. Given that FPR has a relatively smaller impact on system security risks compared to FNR, this trade-off is generally acceptable in practical applications. Nevertheless, the additional computational overhead introduced by FedProx should not be overlooked. Under both Non-IID scenarios, its execution times reached 1399.09 s and 2104.78 s, respectively, i.e., significantly higher than those of FedAvg. The proximal term requires the computation of the divergence between the local and global models during each local optimization step, thereby increasing the computational complexity per training round.

**Figure 9:**
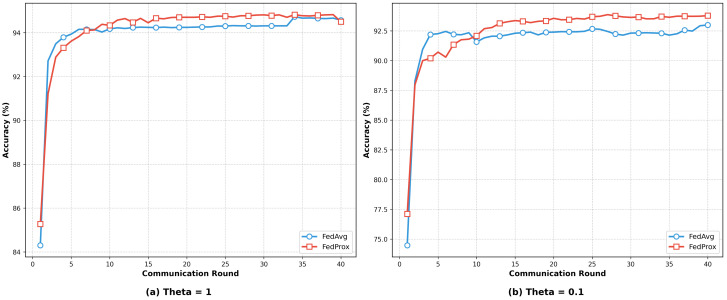
Accuracy comparison of the two aggregation algorithms under different Non-IID scenarios with KD on Edge-IIoT.

Figure 9 illustrates the accuracy trends of the FedAvg and FedProx aggregation algorithms over communication rounds under low- and high-Non-IID data conditions, with the KD mechanism incorporated. When θ=1, due to the relatively consistent data distributions across clients, the local update directions during model training were generally aligned. Consequently, the performance differences between FedAvg and FedProx were minimal. Both methods exhibited a rapid increase in accuracy during the early training stages and gradually converged in the later stages, with their accuracy curves almost overlapping. This indicates that, under low-Non-IID conditions, both algorithms can achieve stable global model training. However, when θ=0.1, the extreme data distribution imbalance poses greater challenges for both algorithms. During the initial stages, FedAvg and FedProx showed similar accuracy improvement trends accompanied by noticeable fluctuations. With an increasing number of communication rounds, the accuracy of FedProx progressively surpassed that of FedAvg and maintained a clear advantage in the later stages. This observation suggests that the introduction of the proximal term in FedProx effectively mitigates the model drift caused by divergent client updates, thereby demonstrating adaptability and model performance in highly Non-IID environments.

In addition, the experimental results on N-BaIoT were also recorded, as presented in Table 11. When θ=1, FedProx outperformed FedAvg in overall performance. Specifically, FedAvg achieved an accuracy and F1-score of 86.49% and 82.16%, respectively, whereas FedProx improved these metrics, achieving 87.70% and 83.52%. In terms of the FNR, FedProx reduced it from 13.51% to 12.30%. However, the FPR exhibited a slight increase from 1.87% to 1.90%. Under θ=0.1, FedProx outperformed FedAvg, achieving an accuracy and F1-score of 83.81% and 78.24%, respectively, compared to the 82.73% and 77.53% for FedAvg, respectively. The FNR of FedProx decreased from 17.27% to 16.19%, while the FPR slightly declined from 2.23% to 2.21%. It is worth noting that, similar to its runtime on Edge-IIoT, FedProx also incurred a significant increase in computational overhead on N-BaIoT.

#### 4.3.5. Impact of Feature Selection on FD-IDS Performance

To assess how feature selection influences the performance of FD-IDS, we performed a comparative analysis of the model’s performance both before and after applying feature selection on Edge-IIoT, as summarized in Table 12. Under the low-Non-IID scenario, the overall performance of FD-IDS remained largely stable after incorporating feature selection. The accuracy remained unchanged at 94.82%, while the F1-score exhibited a slight decrease from 94.44% to 94.35%. In the high-Non-IID scenario, feature selection led to a marginal improvement in model performance, with the accuracy increasing from 93.79% to 93.86% and the F1-score rising from 92.56% to 92.70%. These experimental results indicate that, despite a significant reduction in feature dimensionality, the performance of FD-IDS was not adversely affected; instead, it experienced a slight enhancement. This further validates the effectiveness of the MI method in eliminating redundant and noisy features during the dimensionality reduction process, thereby mitigating interference factors during training.

To comprehensively evaluate the impact of feature selection on training efficiency, we further analyzed the changes in execution time. Table 12 provides the overall runtime of the IDS and the training time of the slowest client. Since the FL setup employed synchronous communication, the global model update in each communication round required waiting for all clients to complete their local training. Consequently, the client with the longest training time was used as the benchmark for comparison. In Table 12, “Client (s)” refers to the cumulative training time of the slowest client over 40 communication rounds. The results in Table 12 show a significant increase in runtime under the high-Non-IID scenario, primarily due to the extreme data imbalance, where certain clients had substantially larger datasets than others, thereby prolonging the overall training process. However, by introducing MI for feature selection, the dimensionality of the features was reduced, effectively lowering computational overhead and shortening training time. Under the low-Non-IID scenario, the total runtime of the IDS decreased from 1538.56 s to 1399.09 s, representing a 9.07% reduction. The training time of the slowest client was reduced from 1191.37 s to 1111.95 s, a 6.67% decrease. Under the high-Non-IID scenario, the total runtime of the IDS decreased from 2247.25 s to 2104.78 s, a reduction of 6.34%, while the training time of the slowest client decreased from 1906.55 s to 1813.38 s, a 4.89% reduction. These results further validate that feature selection slightly enhances model performance while also optimizing training efficiency.

In addition, the experimental results on N-BaIoT were recorded, as presented in Table 13. When θ=1, incorporating feature selection led to a modest performance gain for FD-IDS. The accuracy increased from 87.68% to 87.70%, and the F1-score rose marginally from 83.51% to 83.52%, indicating minimal change. In terms of training efficiency, the total runtime decreased from 522.56 s to 493.30 s, while the training time of the slowest client was reduced from 381.47 s to 366.39 s. When θ=0.1, the introduction of feature selection resulted in a moderate decline in detection performance, although it remained within an acceptable range. Specifically, the accuracy decreased from 84.17% to 83.81%, and the F1-score dropped from 79.35% to 78.24%. Meanwhile, the total runtime was reduced from 547.77 s to 513.66 s, and the training time of the slowest client decreased from 405.23 s to 383.45 s. These results indicate that feature selection also improved training efficiency on the N-BaIoT dataset without causing significant degradation in model performance.

#### 4.3.6. Computational and Communication Overhead Analysis

To evaluate the deployability of the proposed system in real-world IoT scenarios, Table 14 presents a comprehensive analysis of resource utilization, covering critical indicators, such as parameter count, computational complexity, and communication overhead. Given that Edge-IIoT is more recent than N-BaIoT, it was selected as the test dataset in this study. The model was designed with approximately 22,095 trainable parameters, resulting in a compact size of 0.08 MB, thus exhibiting a lightweight architecture suitable for resource-constrained environments.

To assess the computational burden during the local training phase, this study adopted floating-point operations (FLOPs) as a unified metric, quantifying the total number of multiply–accumulate operations involved in each training round. FLOPs are widely recognized as a standard measure of complexity in both the training and inference of neural networks, and they also serve as an indirect indicator of runtime memory requirements due to intermediate activations and temporary data storage—making them particularly relevant for edge deployment considerations. Under the experimental configuration of this work, each client performed two local training iterations per communication round, with an average computational cost of approximately 2.16 × 10^8^ FLOPs per iteration. To assess the practicality of deploying the proposed algorithm on IoT devices, we compared the computational workload of each training iteration with the measured peak performance of commonly adopted Raspberry Pi platforms, as reported in refs. [46,47]. The earlier-generation Raspberry Pi Zero and Raspberry Pi Model 2B exhibited peak performance levels of approximately 3.19 × 10^8^ and 1.47 × 10^9^ FLOPS, respectively, with the latter offering a significant performance improvement over the former. Moreover, the more recently developed Raspberry Pi 4 Model B—widely used in contemporary edge computing evaluations—achieved a peak performance of around 1.15 × 10^10^ FLOPs. A comparative analysis revealed that the computational load required by a single client per training iteration was approximately 2.16 × 10^8^ FLOPs, which was slightly below the peak performance of the Raspberry Pi Zero. In contrast, this workload was substantially lower than the computed capacities of higher-performance edge platforms, such as the Raspberry Pi 2B and Raspberry Pi 4B.

In FL architectures, communication overhead is another critical factor influencing deployment feasibility. As reported in Table 14, the proposed model incurred an aggregate communication volume of approximately 60.69 MB from all clients over 40 communication rounds, corresponding to an average of 1.52 MB per round (with each client transmitting and receiving approximately 0.17 MB of data per round). This level of communication demand falls well within the theoretical bandwidth supported by mainstream wireless standards, such as LTE-M [48].

In summary, the proposed FD-IDS framework demonstrates favorable characteristics for deployment in IoT environments in terms of both computational and communication efficiency.

#### 4.3.7. Performance Comparison Between Federated Learning and Centralized Learning

**Figure 10:**
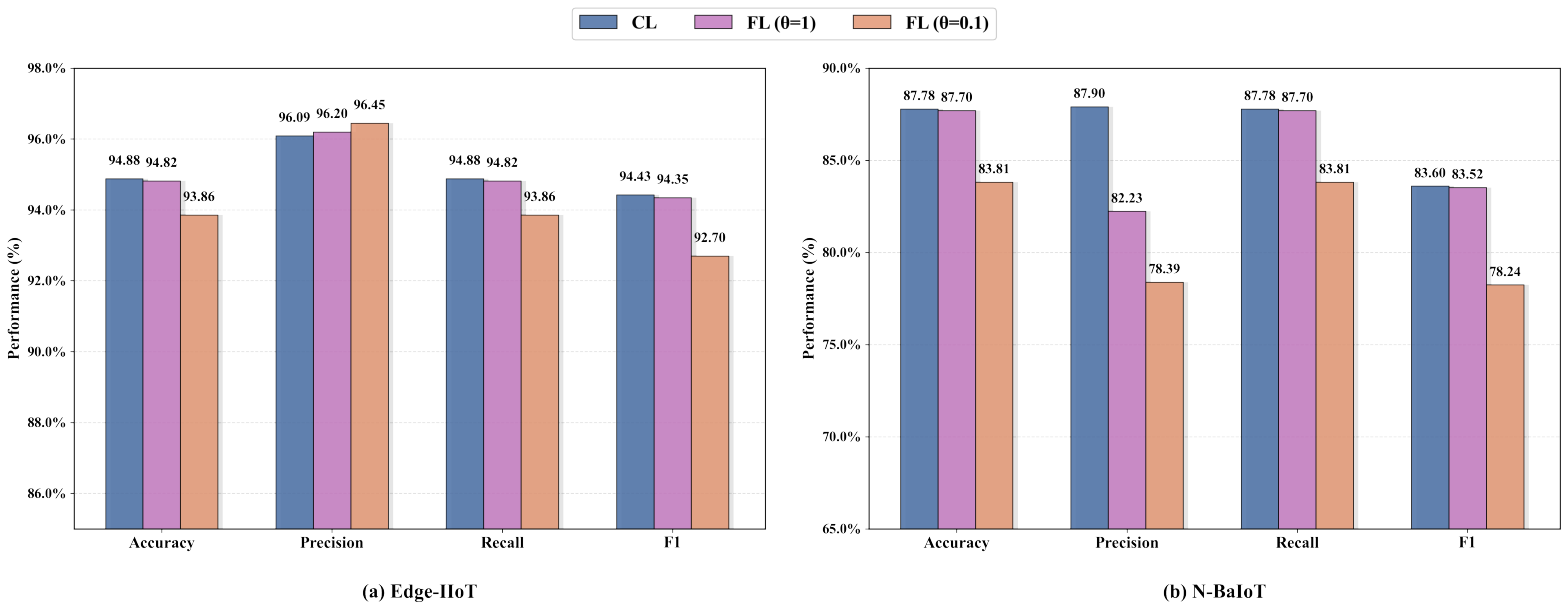
Performance comparison of FD-IDS and CL.

To comprehensively assess the performance of FD-IDS, we conducted a comparative analysis between FD-IDS and CL. In the CL approach, data from all clients are gathered on a central server to create a consolidated training dataset, and the entire model training and updating process is carried out solely on the central server. To ensure the fairness of the experiments and comparability of the results, we employed the same classification model structure and hyperparameters for both CL and FL. The experimental results on Edge-IIoT and N-BaIoT are illustrated in Figure 10. As shown in the figure, both datasets exhibited similar performance trends. Under the low-Non-IID scenario, the performance of FL closely approximated that of CL. This indicates that the FD-IDS method can achieve detection performance comparable to CL when the data distribution differences across clients are relatively small. On the other hand, while high-Non-IID data introduced certain performance challenges for FL, which was more pronounced in the N-BaIoT dataset, FD-IDS still demonstrated reliable performance, maintaining a reasonable gap compared to CL. Although CL exhibited slightly superior performance, it requires transferring all raw data to the central server, which poses significant privacy risks. Additionally, CL is heavily reliant on the central server. As the data scale increases, the central server faces substantial storage and computational demands, potentially creating severe performance bottlenecks in practical applications. In contrast, FD-IDS effectively mitigates these challenges by leveraging FL.

#### 4.3.8. Performance Comparison Between FD-IDS and Existing Methods

To thoroughly evaluate the effectiveness of our approach, we compared it with existing methods that were evaluated on the Edge-IIoT dataset in a multi-class classification task. The results of the comparison are displayed in Table 15. Ferrag et al. [26] developed the Edge-IIoT dataset and conducted FL experiments using a DNN model in both IID and Non-IID scenarios, achieving accuracies of 93.89% and 91.74%, respectively. Rashid et al. [27] explored the performance of CNNs and RNNs. Under IID and Non-IID conditions, the classification accuracies of the CNN were 91.27% and 90.73%, while the RNN achieved accuracies of 92.37% and 91.87%. Aouedi et al. [28] proposed an ensemble learning framework, F-BIDS, based on FL, which achieved a classification accuracy of 89.91%. Additionally, Benameur et al. [33] combined FL with KD techniques, reporting accuracies of 82.4% with a CNN-LSTM model, as well as 82.35% and 82.09% for the CNN and DNN models, respectively. In contrast to these methods, our approach focuses on performance optimization in Non-IID scenarios. By incorporating a proximal term and KD, we improve the model’s accuracy under complex data distribution conditions.

However, we acknowledge that, although our study and the aforementioned methods were conducted on the same dataset, the direct performance comparisons presented in Table 15 should be interpreted with caution. This is primarily due to notable differences in experimental configurations across studies, such as the simulation of Non-IID conditions, the number of clients, and the data preprocessing procedures. These variations can substantially influence the final performance metrics. Therefore, we do not consider the performance advantages of our method in Table 15 as definitive evidence of superiority over existing approaches, but rather as indicative results under the utilized experimental settings.

To further validate the effectiveness of FD-IDS, we conducted performance comparisons with existing approaches under the same experimental conditions on the Edge-IIoT and N-BaIoT datasets. Table 16 presents the experimental results on Edge-IIoT, comparing the performance of the proposed FD-IDS with that of FedAvg-only, FedProx-only, and SIM-FED under the settings of θ=1 and θ=0.1. It is important to note that methods such as FedAvg and FedProx do not incorporate KD techniques. The experimental results demonstrate that, under low-Non-IID conditions, FD-IDS outperforms the compared approaches in terms of accuracy and F1 score. Under high-Non-IID conditions, the advantage of FD-IDS becomes even more pronounced as it outperforms all other methods across all evaluation metrics. Similarly, Table 17 presents the experimental results on N-BaIoT, revealing a similar trend. When θ=1, the FD-IDS method outperforms the compared methods in terms of accuracy and F1 score. When θ=0.1, FD-IDS surpasses all other methods across all evaluation metrics.

### 4.4. Discussion

Through the experiments and analyses presented in the preceding sections, this study comprehensively evaluated the effectiveness of FD-IDS in IoT settings and validated the effectiveness of the proposed method across multiple aspects. To address the model drift caused by Non-IID data distributions in FL, this study integrated a proximal term and KD to collaboratively optimize the model at both global and local levels. This enables the model to maintain superior detection performance, even under scenarios with high data heterogeneity. Furthermore, by employing MI-based feature selection, the proposed method preserves critical features while reducing feature dimensionality, thereby decreasing computational overhead and further enhancing system training efficiency and detection capability. In addition, the analysis of computational and communication overhead reveals that FD-IDS exhibits lightweight characteristics, making it suitable for deployment on commonly used edge devices, such as the Raspberry Pi. Its communication demands also remain well within the bandwidth constraints of mainstream wireless standards. Finally, experimental results demonstrate that the performance of FD-IDS under FL closely approximates its performance under CL, which substantiates the efficiency and practicality of FL approaches while simultaneously preserving data privacy.

Despite its strengths, the proposed method has certain limitations that warrant further improvement and optimization in future research. Firstly, while the integration of a proximal term and KD improves model performance, it also increases the computational burden on clients. This may pose challenges for resource-constrained IoT devices. Future research will aim to optimize the distillation process by designing lightweight distillation mechanisms that reduce computational complexity while maintaining performance improvements. Secondly, we recognize that the current work has not explicitly addressed the issue of concept drift. Specifically, this study assumed a static data distribution and did not account for the dynamic nature of network environments and attack patterns in IoT applications. In practice, intrusion behaviors and normal traffic characteristics within IoT systems often evolve over time, exhibiting significant concept drift, which may lead to model performance degradation or even failure in long-term deployments. Without dynamic adaptation capabilities, IDSs may struggle to effectively respond to continuously changing threat scenarios. To ensure long-term robustness and adaptability, future research will explore mechanisms that enable FD-IDS to continuously adapt to concept drift and evolving attack patterns, including—but not limited to—online learning, incremental learning, and continual learning strategies. Thirdly, although the effectiveness of FD-IDS has been demonstrated using the Edge-IIoTset and N-BaIoT datasets—which include a representative range of common IoT attack scenarios—relying solely on these two datasets may fail to capture the full heterogeneity present in IoT. To further validate the adaptability of the proposed method, future research will consider conducting comprehensive evaluations on multiple publicly available IoT intrusion detection datasets. Finally, this study assumed that all client nodes were secure, and it did not account for potential security threats. However, in practical scenarios, FL may be vulnerable to various malicious behaviors, such as poisoning attacks and membership inference attacks. These threats can not only lead to significant degradation in global model performance, but also pose severe risks of user privacy breaches. Therefore, future research will focus on developing a more robust and secure federated intrusion detection framework. Key directions include the incorporation of adversarial defense mechanisms, such as client trust evaluation and weighted aggregation strategies, as well as the integration of privacy-preserving techniques, like differential privacy, to effectively mitigate the risk of sensitive information leakage. These enhancements aim to ensure the reliability and practicality of the system under non-ideal or even adversarial conditions.

## 5. Conclusions

This paper proposes an IDS based on FL, termed FD-IDS, which conducts an in-depth investigation and introduces effective improvements to address the widespread issue of Non-IID data in IoT environments. First, the MI-based feature selection method is adopted to filter out redundant features from high-dimensional data, thereby reducing the computational overhead while enhancing the representation capability of features. Second, to tackle the performance degradation and global model drift caused by Non-IID data distributions in FL environments, this work introduces the FedProx aggregation strategy. By adding a proximal term during optimization, the method effectively suppresses the instability and performance degradation stemming from data heterogeneity across devices. In addition, a KD-based training mechanism is proposed, where the global model acts as a teacher to supervise the training of local models. This global–local collaborative approach enhances the overall model performance and improves the detection accuracy in heterogeneous data environments. Comprehensive experiments were performed utilizing both Edge-IIoT and N-BaIoT. The results demonstrate that FD-IDS achieves outstanding performance in Non-IID scenarios. Moreover, it exhibits favorable adaptability in terms of computational and communication overhead, highlighting its potential for deployment in IoT environments.

Future research will focus on further optimizing FD-IDS. First, efforts will be directed toward designing more efficient distillation strategies to reduce computational complexity, thereby alleviating the burden on resource-constrained IoT devices. Second, future work will focus on enhancing FD-IDS’s capability to adapt to concept drift and respond to evolving attack patterns in long-term deployments. Third, we plan to conduct evaluations on multiple publicly available IoT intrusion detection datasets to validate the generalization capability of the proposed method under diverse data distributions and attack scenarios. Finally, subsequent work will investigate defense mechanisms to counter potential threats, such as poisoning attacks from malicious clients, thereby enhancing the system’s reliability in adversarial environments.

## Figures and Tables

**Table 2:** Hyperparameter search space.

Hyperparameter	Candidate Values
Batch Size	[64, 128, 256]
Learning Rate	[0.001, 0.01, 0.1]
Regularization (μ)	[0.001, 0.01, 0.1]
Distillation Weight (λ)	[0.3, 0.5, 0.7]
Proximal Term Weight (β)	[0.05, 0.1, 0.2]
Temperature (*T*)	[1, 3, 5]

**Table 3:** Hyperparameter settings.

Hyperparameter	Settings
Batch Size	128
Optimizer	Adam
Learning Rate	0.001
DNN Activation Function	Relu
Loss Function	Cross-Entropy Loss
Regularization (μ)	0.01
Distillation Weight (λ)	0.5
Proximal Term Weight (β)	0.1
Temperature	3
Local Epoch	2
Communication Round	40

**Table 4:** Confusion matrix.

	Predicted Attack	Predicted Normal
**Actual Attack**	True Positive (TP)	False Negative (FN)
**Actual Normal**	False Positive (FP)	True Negative (TN)

**Table 5:** Performance evaluation of FD-IDS in different Non-IID scenarios.

Concentration Parameter	1st Round			40th Round		
	**B**	**W**	**G**	**B**	**W**	**G**
Edge-IIoT						
θ=1	91.94	88.75	85.27	94.65	92.50	94.82
θ=0.1	86.08	7.32	77.12	93.50	88.25	93.86
N-BaIoT						
θ=1	72.87	55.10	53.21	87.69	86.38	87.70
θ=0.1	55.85	10.38	38.58	83.25	78.45	83.81

**B**: **B**est client accuracy; **W**: **W**orst client accuracy; and **G**: **G**lobal model accuracy.

**Table 6:** Performance comparison of the different KD intervals in different Non-IID scenarios (Edge-IIoT).

KD Intervals	Accuracy	Precision	Recall	F1
Low Non-IID (θ=1)				
Periodic KD	94.71	95.38	94.71	94.25
End-of-Training KD	94.70	95.10	94.70	94.42
Round-wise KD	94.82	96.20	94.82	94.35
High Non-IID (θ=0.1)				
Periodic KD	93.21	94.50	93.21	91.94
End-of-Training KD	92.97	93.31	92.97	91.58
Round-wise KD	93.86	96.45	93.86	92.70

**Table 7:** Performance comparison of the different KD intervals in different Non-IID scenarios (N-BaIoT).

KD Intervals	Accuracy	Precision	Recall	F1
Low Non-IID (θ=1)				
Periodic KD	86.28	79.91	86.28	81.96
End-of-Training KD	86.25	79.87	86.25	81.93
Round-wise KD	87.70	82.23	87.70	83.52
High Non-IID (θ=0.1)				
Periodic KD	81.53	74.29	81.53	76.00
End-of-Training KD	80.72	76.15	80.72	76.72
Round-wise KD	83.81	78.39	83.81	78.24

**Table 8:** Performance comparison of an IDS with and without distillation under high- and low-Non-IID scenarios on Edge-IIoT (FedProx strategy).

KD	Accuracy	Precision	Recall	F1	FPR	FNR	Runtime (s)
Low Non-IID (θ=1)							
No	94.74	95.98	94.74	94.17	0.14	5.26	1312.41
Yes	94.82	96.20	94.82	94.35	0.13	5.18	1399.09
High Non-IID (θ=0.1)							
No	91.89	94.67	91.89	90.71	0.18	8.11	1948.90
Yes	93.86	96.45	93.86	92.70	0.21	6.14	2104.78

**Table 9:** Performance comparison of an IDS with and without distillation under high- and low-Non-IID scenarios on N-BaIoT (FedProx strategy).

KD	Accuracy	Precision	Recall	F1	FPR	FNR	Runtime (s)
Low Non-IID (θ=1)							
No	87.56	82.30	87.56	83.38	1.91	12.43	469.62
Yes	87.70	82.23	87.70	83.52	1.90	12.30	493.30
High Non-IID (θ=0.1)							
No	81.51	76.93	81.51	77.17	2.21	18.49	479.13
Yes	83.81	78.39	83.81	78.24	2.21	16.19	513.66

**Table 10:** Performance of the different aggregation algorithms under low- and high-Non-IID scenarios with KD on Edge-IIoT.

Aggregation Algorithms	Accuracy	Precision	Recall	F1	FPR	FNR	Runtime (s)
Low Non-IID (θ=1)							
FedAvg	94.73	96.04	94.73	94.33	0.13	5.27	902.61
FedProx	94.82	96.20	94.82	94.35	0.13	5.18	1399.09
High Non-IID (θ=0.1)							
FedAvg	92.99	93.81	92.99	92.08	0.18	7.01	1268.78
FedProx	93.86	96.45	93.86	92.70	0.21	6.14	2104.78

**Table 11:** Performance of the different aggregation algorithms under low- and high-Non-IID scenarios with KD on N-BaIoT.

Aggregation Algorithms	Accuracy	Precision	Recall	F1	FPR	FNR	Runtime (s)
Low Non-IID (θ=1)							
FedAvg	86.49	80.11	86.49	82.16	1.87	13.51	332.23
FedProx	87.70	82.23	87.70	83.52	1.90	12.30	493.30
High Non-IID (θ=0.1)							
FedAvg	82.73	78.39	82.73	77.53	2.23	17.27	340.99
FedProx	83.81	78.39	83.81	78.24	2.21	16.19	513.66

**Table 12:** Performance comparison of FD-IDS before and after MI on Edge-IIoT.

MI	Accuracy	Precision	Recall	F1	Runtime (s)	Client (s) *
Low Non-IID (θ=1)						
Before	94.82	95.86	94.82	94.44	1538.56	1191.37
After	94.82	96.20	94.82	94.35	1399.09	1111.95
High Non-IID (θ=0.1)						
Before	93.79	93.54	93.79	92.56	2247.25	1906.55
After	93.86	96.45	93.86	92.70	2104.78	1813.38

* denotes the cumulative training time of the slowest client across 40 rounds.

**Table 13:** Performance comparison of FD-IDS before and after MI on N-BaIoT.

MI	Accuracy	Precision	Recall	F1	Runtime (s)	Client (s) *
Low Non-IID (θ=1)						
Before	87.68	82.75	87.68	83.51	522.56	381.47
After	87.70	82.23	87.70	83.52	493.30	366.39
High Non-IID (θ=0.1)						
Before	84.17	78.32	84.17	79.35	547.77	405.23
After	83.81	78.39	83.81	78.24	513.66	383.45

* denotes the cumulative training time of the slowest client across 40 rounds.

**Table 14:** Summary of the computational and communication costs in FD-IDS.

Metric	Value	Description
Number of Model Parameters	22,095	Total number of trainable parameters in the model.
Model Size (MB)	0.08	Storage space occupied by the model file on the device.
Total FLOPs	1.56 × 10^11^	Total computation across all clients during 40 communication rounds.
Average FLOPs per Round	2.16 × 10^8^	Computation cost per client per local training iteration per round.
Total Communication Volume (MB)	60.69	Total upload + download volume across all clients over 40 rounds.
Communication Volume per Round (MB)	1.52	Upload + download volume across all clients per round.
Per-Client Communication per Round (MB)	0.17	Upload + download volume per client per round.

**Table 15:** Performance comparison of FD-IDS and existing methods.

Ref	Algorithm	Data Distribution	Accuracy
[26]	DNN	IID	93.89
		Non-IID	91.74
[27]	CNN	IID	91.27
		Non-IID	90.73
	RNN	IID	92.37
		Non-IID	91.87
[28]	F-BIDS	**Ø**	89.91
[33]	DNN	**Ø**	82.09
	CNN	**Ø**	82.35
	CNN-LSTM	**Ø**	82.4
Our method	FD-IDS (θ=1)	Low Non-IID	94.82
	FD-IDS (θ=0.1)	High Non-IID	93.86

**Ø** indicates that the corresponding study did not mention this information.

**Table 16:** Performance comparison of FD-IDS and existing methods under the same experimental setup (Edge-IIoT).

Ref	Algorithm	Accuracy	Precision	Recall	F1
Low Non-IID (θ=1)					
[11]	FedAvg-only	94.65	96.02	94.65	94.13
[34]	FedProx-only	94.74	95.98	94.74	94.17
[29]	SIM-FED	94.68	96.69	94.68	94.03
Our method	FD-IDS	94.82	96.20	94.82	94.35
High Non-IID (θ=0.1)					
[11]	FedAvg-only	90.92	95.29	90.92	88.93
[34]	FedProx-only	91.89	94.60	91.89	90.88
[29]	SIM-FED	91.21	89.48	91.21	89.78
Our method	FD-IDS	93.86	96.45	93.86	92.70

**Table 17:** Performance comparison of FD-IDS and existing methods under the same experimental setup (N-BaIoT).

Ref	Algorithm	Accuracy	Precision	Recall	F1
Low Non-IID (θ=1)					
[11]	FedAvg-only	86.50	85.48	86.50	82.18
[34]	FedProx-only	87.56	82.30	87.56	83.38
[29]	SIM-FED	87.46	86.73	87.46	83.29
Our method	FD-IDS	87.70	82.23	87.70	83.52
High Non-IID (θ=0.1)					
[11]	FedAvg-only	80.43	77.31	80.43	76.68
[34]	FedProx-only	81.51	76.93	81.51	77.17
[29]	SIM-FED	74.63	74.80	74.63	69.48
Our method	FD-IDS	83.81	78.39	83.81	78.24

## Data Availability

This study used two publicly available datasets (both accessed on 9 June 2025): the Edge-IIoTset dataset (https://ieee-dataport.org/documents/edge-iiotset-new-comprehensive-realistic-cyber-security-dataset-iot-and-iiot-applications), and the N-BaIoT dataset (http://archive.ics.uci.edu/ml/datasets/detection_of_IoT_botnet_attacks_N_BaIoT).
